# EZH2 Regulates Cofilin Activity and Colon Cancer Cell Migration by Targeting ITGA2 Gene

**DOI:** 10.1371/journal.pone.0115276

**Published:** 2014-12-30

**Authors:** Angelo Ferraro, Themis Boni, Alexander Pintzas

**Affiliations:** Laboratory of Signal Mediated Gene Expression, Institute of Biology, Medicinal Chemistry and Biotechnology, National Hellenic Research Foundation, 48, Vas. Constantinou Avenue, 11635, Athens, Greece; Deutsches Krebsforschungszentrum, Germany

## Abstract

Reorganization of cytoskeleton via actin remodeling is a basic step of cell locomotion. Although cell migration of normal and cancer cells can be stimulated by a variety of intra- and extra-cellular factors, all paths ultimate on the regulation of cofilin activity. Cofilin is a small actin-binding protein able to bind both forms of actin, globular and filament, and is regulated by phosphorylation at Serine 3. Following phosphorylation at serine 3 cofilin is inactive, therefore cannot bind actin molecules and cytoskeleton remodeling is impaired. The histone methyltransferase EZH2 is frequently over expressed in many tumour types including colorectal cancer (CRC). EZH2 over activity, which results in epigenetic gene-silencing, has been associated with many tumour properties including invasion, angiogenesis and metastasis but little is known about the underneath molecular mechanisms. Herein, we report that EZH2 is able to control cofilin activity and consequently cell locomotion of CRC cell lines through a non-conventional novel axis that involves integrin signaling. Indeed, we show how genetic and pharmacological inhibition (DZNep and GSK343) of EZH2 function produces hyper phosphorylation of cofilin and reduces cell migration. We previously demonstrated by chromatin immuno-precipitation that Integrin alpha 2 (ITGα2) expression is regulated by EZH2. In the present study we provide evidence that in EZH2-silenced cells the signaling activity of the de-repressed ITGα2 is able to increase cofilin phosphorylation, which in turn reduces cell migration. This study also proposes novel mechanisms that might provide new anti-metastatic strategies for CRC treatment based on the inhibition of the epigenetic factor EZH2 and/or its target gene.

## Introduction

Tumour cell migration is essential for metastatic capability acquisition [1], [2]. Migration competence requires activation of signaling pathways that converge into actin polymerization and de-polymerization which drive the formation of special cell-membrane protrusions such as lamellipodia, invadopodia and filopodia. Actin dynamics is regulated by numerous actin-binding proteins, among them actin-depolymerizing factor (ADF), cofilin-1 (non-muscle type) and cofilin-2 (muscle type) are those that allow cell locomotion through the above described membrane structures [3], [4]. Since cofilin-1 is the most ubiquitous form of actin-binding proteins, in the present study we analyzed only this type, and herein we refer to it as cofilin. Cofilin activation is a key step for tumour cell migration [5], [6], although, most likely, it is the overall activity of the cofilin-regulating pathways that drive the motility of cancer cells [4]. Several mechanisms of cofilin regulation are known [3], [4]. The best characterized are through phosphorylation at Serine 3 (p-cofilin) by LIM kinase 1 and 2 (LIMK1, LIMK2) [7], by testicular protein kinase 1 and 2 (TESK1, TESK2) [8], and by de-phosphorylation at serine 3 by phosphatase slingshot (SSH) and chronophin [9], [10]. Phosphorylation at serine 3 inactivates cofilin, preventing its binding to the major substrates globular-actin (G-actin) and filament-actin (F-actin) [11], whereas de-phosphorylation at Serine 3 allows substrate binding and F-actin severing. Several signaling mechanisms control cofilin functions and consequently cell-shape and -locomotion. They range from Rho family small GTPases acting on LIMKs, to integrin-mediated signaling acting on TESK1/2 [12]. SSH phosphatase can be activated by F-actin, 14-3-3 proteins, PKDs and by PLCβ/PI3Kγ-GSK3 signaling pathway [12].

Enhancer of Zeste Homolog 2 (EZH2) belongs to the polycomb group family [13] and is the catalytic subunit of the histone methyltransferase complex called Polycomb Repressive Complex 2 (PRC2). PRC2 binds to target gene promoters for epigenetic repression via di- or trimethylation of histone H3 at lysine 27 residue (H3K27me2-3). EZH2 over-expression is associated, in many aggressive tumours [14], [15], with poor prognosis [16] and presence of distant metastasis in colorectal cancer (CRC) [17]. EZH2 downregulation can reduce growth of invasive breast carcinoma [18], tumour angiogenesis [19] and *in vitro* cell migration/invasion of CRC cell lines [17].

The well defined architecture of epithelial tissues, such as colon mucosa, largely depends on the expression of specific cell surface adhesion molecules such as integrins, that interact with extracellular matrix (ECM) and neighboring cells. The mammalian genomes encode 18 α- and 8 β-integrin subunits, that in combination produce 24 different integrin (α-β-) heterodimers with no redundant functions ranging from extracellular collagen receptors to signal transduction mediators [20]. Changes in integrin expression occur in many cancer types and whether integrins act only as tumour enhancers or tumour suppressors is still debated. ITGα2 forms heterodimers with ITGβ1 -α2β1- and interacts with ECM’s collagen fibers [20]. It has been reported that integrin α2β1 is able to block cell proliferation [21]–[23] and to suppress metastasis in mice and humans [24]. In prostate cancer, ITGα2 down regulation has been proposed as potential tumour marker [25]. We recently proved that EZH2 epigenetically represses ITGα2 expression [17]. Interestingly, during the characterization of the newly established EZH2-silenced cell line (named HCT-shEZ-2), a remarkable hyper-phosphorylation of cofilin in HCT-shEZ-2 as compared to HCT116 parental cells was detected.

Although cofilin function has been described in tumour cell biology, regulatory networks that integrate epigenetic factors, deregulation of signal transduction pathways and cofilin activity are poorly described. Here we show that the histone methyltransferase EZH2 controls cofilin phosphorylation, and consequently cell locomotion, through a novel path that involves ITGα2 and its signal-transduction activity.

## Materials and Methods

### Cell culture

Caco-2, DLD-1, HT-29, HCT116 and RKO cell lines were maintained in DMEM medium, containing 10% fetal bovine serum (FBS), 1X penicillin, 1X streptomycin, and 2 mM L-glutamine. Cells were incubated at 37°C, 5% CO_2_ in humidified atmosphere. All cell lines used in the study have been purchased from American Type Culture Collection (ATCC). The cloning procedure for HCT-shEZ-2 cell line is described in Ferraro et. al. [17].

### Western blot and antibodies

Total protein was extracted with 60 µL RIPA lysis buffer and Western blotting was performed according to standard protocols. Blots were incubated overnight at 4°C with the appropriate primary antibodies. Antibodies used: EZH2 BD Bioscience, USA code 612667; from Millipore, USA H3K27me3 code 07-449; RAC-1 code 05-389; from Santa Cruz, Germany: H3 total code sc-8654; Tubulin code sc-8035; ITGα2 code sc-74466; RhoA code sc-418; ROCK1 code sc-17794; Cdc42 code sc-87; p-cofilin (Serine 3) code sc-12912-R; cofilin sc-33779; TESK1 code sc-366681 (to increase the specificity of TESK1 antibody in WB assays nitrocellulose blotting membranes were cut horizontally between ∼60 and ∼80 kDa). Protein extracts have been prepared from at least two independent experiments and blots repeated at least twice.

### Confocal microscopy

Cells used for immunofluorescence assays were fixed with methanol/acetone solution (8∶1), washed, permeabilized with 0.3% Triton-X-100, and blocked with 5% BSA prior to incubation with primary antibodies or with Alexa fluor 546 phalloidin (Invitrogen USA, code A22283). Nuclei were counterstained with Hoechst.

### Inhibitors and siRNA transfection

ROCK-1 inhibitor (Y-27632 Sigma, USA code Y0503-1MG) was used at a final concentration of 30 µM for 24 hours. EZH2 inhibitors (3-Deazaneplanocin A (DZNep) Santa Cruz, Germany code sc-351856; GSK-343 Active Biochemicals, Hong Kong code A-1416) were used at a final concentration of 1, 3 and 5 µM for 48–72 hours. For siITGα2 transfection, cells were seeded in 6-well plates and transfected with Lipofectamnine 2000, siEZH2_4 and siEZH2_7 (Quiagen, Germany codes SI00063973, SI02665166) siITGα2 (Quiagen, Germany codes SI02664081, SI02664088) and siRhoA (Dharmacon, USA code L-003860-00-0005) have been used at a final concentration of 50 µM according to the manufacturer’s instructions.

### Three-dimensional culture

For three-dimensional culture Matrigel (BD, Bioscience) was used. Poly-lysine pre-coated cover slips were put in 24-well plate. Matrigel was diluted with cold complete DMEM medium to a final concentration of 50%, 200 µl were deposited on each well containing cover slips and the plate was incubated at 37°C for 15′. 0.5×10^3^ cells was diluted in 100 µl of cold DMEM and mixed with 100 µl of 100% Matrigel. The resulting 200 µl were added to the wells with pre-warmed Matrigel. Plate was incubated in a humidified atmosphere at 37° with 5% CO_2_ for 3 days.

### Migration assay

Cells were trypsinized, washed with medium containing 1% FBS, and counted. 10^5^ cells were plated into upper chamber of an 8 µm-pore Transwell filter (Corning), mounted in a 24-well dish containing 10% FBS medium. Filters were pre-coated with fibronectin. Cells were allowed to migrate at 37°C, 5% CO_2_ for 36–40 h, fixed with methanol and stained with 0.1% w/v crystal violet. Underside of filters was observed with 40X objective and migrating cells were determined in each well. Experiments were performed in duplicate and repeated twice. For migration assay coupled with siITGα2 transfection, cells were trypsinized 24 hours after transfection, seeded on transwell and incubated at 37°C for other 24 hours. For migration assay coupled with ROCK1 inhibitor, cells were seeded on transwell in the presence of the inhibitor and incubated at 37°C for another 24 hours.

### Globular (G-) and Filament (F-) actin assay

80–85% confluent cells were washed directly on 15 cm petri dishes twice with PBS at room-temperature, scraped into PBS and pelleted. Cell pellets were resuspended in 800 µl of 37°C lysis buffer (1 mM ATP, 50 mM PIPES pH 6.9, 50 mM NaCl, 5 mM MgCl2, 5 mM EGTA, 5% vol/vol glycerol and 0.1% vol/vol for: Nonidet P-40, Tween 20, and Triton X-100) supplemented with protease inhibitors. Cells were homogenized by gently pipetting 10 times up and down and incubated at 37°C for 30 min. Samples were centrifuged at 16000 g for 75 min at 22–25°C. Supernatants were removed for determination of G-actin. Pellets containing F-actin were mixed with 800 µl of RIPA buffer and sonicated on ice for 5 min (30 sec. ON/30 sec OFF, max power) using Bioruptor ultrasonicator (Diagenode, Belgium) to dissolve them. Twenty microliters of each fraction were mixed with SDS-loading buffer, denatured for 8 minutes at 95°C and loaded on polyacrylamide gel. Actin detection was performed using an anti-actin monoclonal antibody (code sc-8432) purchased from Santa Cruz, Germany.

### RNA Extraction/Reverse Transcription and Real Time-PCR

Total RNA isolation from cultured cells was performed using the Trizol reagent (Invitrogen, Karlsruhe, Germany). Reverse transcription was carried out from 3.0 µg of purified RNA using the SuperScript Reverse Transcriptase (Invitrogen, Karlsruhe, Germany) following the manufacturer's instructions.

Real-time quantification at the mRNA level was carried out in 96-well PCR plates using a Bio-Rad iCycler and the iQ5 Multicolor real-Time PCR detection system (Bio-Rad, Hercules CA, USA). Each reaction contained 1× iQ SYBR Green Supermix (Bio-Rad, Hercules CA, USA) and 150 nmol/L of each primer. All genes were tested in triplicates. Results were analyzed on the iCycler software. Values were normalized to GAPDH. Primers used were the following: glyceraldehyde-3-phosphate dehydrogenase (GAPDH): GAAGGTGAAGGTCGGAGT (Fw) and CATGGGTGGAATCATATTGGAA (Rv); TESK1: GCCCTGACACACAATCAG (Fw) and AAGAGGTCTGACCGGCTTC (Rv).

### GST pull-down assay

For Rac1-GTP and CDC42-GTP GST pull-down assay, cells were cultured in 10 cm petri dishes and cell lysates were prepared with lysis buffer used for western blotting. 50 µg of the identical protein extract were incubated with GST-PAK (p21-activated kinase) to glutathione agarose beads for 1 hour by rotating at 4°C and beads were washed 4 times in wash buffer (50 mM Tris-HCl (pH 7.4), 150 mM NaCl, 10 mM MgCl2, 1% (v/v) Triton X-100, 1 mM dithiothreitol (DTT), 10 µg/ml aprotinin, 10 µg/ml leupeptin and 0.2 mM PMSF).

## Results

### Cofilin phosphorylation increases upon genetic silencing and pharmacological inhibition of EZH2 in CRC cell lines

**Figure 1:**
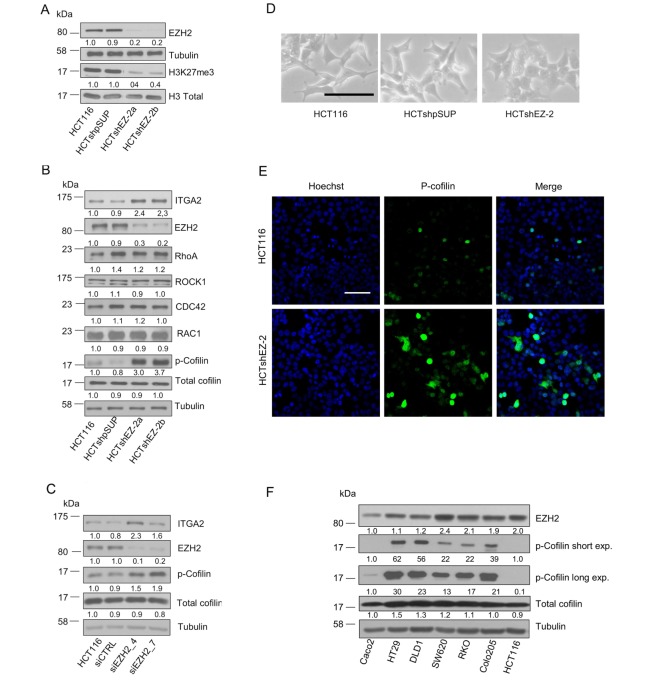
Low EZH2 expression correlates with a remarkable hyper-phosphorylation of cofilin. A. Protein expression and global methyltransferase activity of EZH2 in HCT116 and in HCTshEZ-2 stable clones as well as in HCTshpSUP mock control cells analyzed by WB. B. Protein expression analysis of Rho GTPase family components and ITGα2 in HCT116, HCTshEZ-2 and HCTshSUP cells. C. Transient depletion of EZH2 with two different siRNA oligos increases cofilin phosphorylation similarly to stable silencing. D. Phase contrast microscopy pictures of HCT116, HCTshEZ-2 and HCTshSUP cell-phenotypes, scale bar 150 µm. E. Representative pictures of confocal fluorescent microscope in HCT116 and HCTshEZ-2 cells where p-cofilin was stained (green). Nuclei have been counterstained with hoechst (blue). Scale bar size 150 µm. F. EZH2 protein levels are compared with cofilin and p-cofilin levels in 7 CRC cell lines. Numbers below blots indicate band quantification which was performed using a dedicated software and Tubulin expression as reference loading control. All WB experiments have been repeated at least three times, representative blots and quantifications are presented.

We previously transfected HCT116 colon carcinoma cells with a short hairpin RNA (shRNA) vector carrying a stem loop oligonucleotide against EZH2 mRNA. The stable shEZH2 clones (HCTshEZ-2) display very low amount of EZH2 protein as well as low trimethylation at lysine 27 of histone H3 (H3K27me3), the EZH2-specific substrate (Fig. 1A). We reported that HCTshEZ-2 cells show reduced migratory and invasive capacity as compared with parental and mock transfected (HCTshpSUP) HCT116 cells [17]. To identify potential factors that might control cell locomotion of HCTshEZ-2 cells, protein expression analysis of factors involved in the regulation of cytoskeleton dynamics such as RhoA, CDC42, ROCK1, RAC1, Cofilin and p-cofilin (downstream effectors of Rho GTPase pathway) have been analyzed by western blot (WB). As reported in Fig. 1B, silencing of EZH2 did not affect protein expression levels of none of the analyzed actin-modulating factors. Conversely, a remarkable hyper-phosphorylation at Serine 3 of the small actin-binding protein cofilin was detected (Fig. 1B). Furthermore, in HCTshEZ-2 cells silencing of EZH2 did not change the GTPase activity of CDC42 and RAC1 that was evaluated using GST-PAK pull down assay (S1 Fig.). We also investigated indirectly the potential activation of RhoA in HCTshEZ-2 cells. By silencing RhoA with siRNA technology we found that p-cofilin level remained the same across all cell lines (HCT116, HCTshpSUP and HCTshEZ-2). Therefore, depletion of EZH2 expression did not affect RhoA activity and consequently cofilin phosphorylation (S2A Fig.). To prove that the observed hyper-phosphorylation of cofilin is not an off-target effect of the cloning procedure, EZH2 was transiently depleted with two different siRNAs oligos in HCT116 and RKO cells and 48 h after levels of p-cofilin were analyzed by WB. As shown in Fig. 1C for HCT116 and in S2B Fig. for RKO, transient EZH2 silencing resulted in a significant increasing of p-cofilin as well as of ITGα2 levels in both cell lines.

The effect of cofilin inactivation on phenotype and growth properties of HCTshEZ-2 cells is well evident in standard two-dimensional culture. Indeed, in HCTshEZ-2 cells the number of cell-membrane protrusions is poor, less prominent and the elongated shape of control HCT116 cells is lost, being HCTshEZ-2 cells more round and flat (Fig. 1D). Using confocal microscopy and fluorescent immunoassay to stain p-cofilin, we confirmed that inactive p-cofilin is increased in HCTshEZ-2 cells compared to the parental HCT116 cells and that p-cofilin is mainly localized in the nucleus of HCT116, whereas in HCTshEZ-2 cells p-cofilin is localized both in the nucleus and in the cytoplasm (Fig. 1E).

To verify whether the relation between EZH2 and p-cofilin is a general characteristic of CRC cell lines, we analyzed cofilin, p-cofilin and EZH2 expression levels in 7 CRC cell lines. In these 7 CRC cell lines a previous comparison between EZH2 protein and global levels of its substrate H3K27me3 showed that cells over-expressing EZH2 (HCT116, RKO and SW620) also present high levels of H3K27me3 (data not shown, see ref. [17]). Interestingly, cell lines with high EZH2 protein levels have shown almost undetectable (HCT116) or low (SW620 and RKO) p-cofilin as compared to the other studied cell lines, with the exception of Caco-2 intermediate adenoma cell line (Fig. 1F). By contrast, levels of the cofilin protein (total-cofilin) were substantially the same across the 7 cell lines studied. In order to quantify the correlation between EZH2 expression and cofilin phosphorylation in all CRC cell lines, with the exception of Caco2, the absolute intensity of EZH2 WB bands of Fig. 1F has been plotted versus the absolute intensity of p-cofilin WB bands using a scatter chart. A significant negative correlation coefficient (*R* = −0.7532) of the straight line obtained with the data-points indicates that p-cofilin levels are inversely correlated with respect to EZH2 levels in 6 out of 7 CRC cell lines (S3 Fig.).

**Figure 2:**
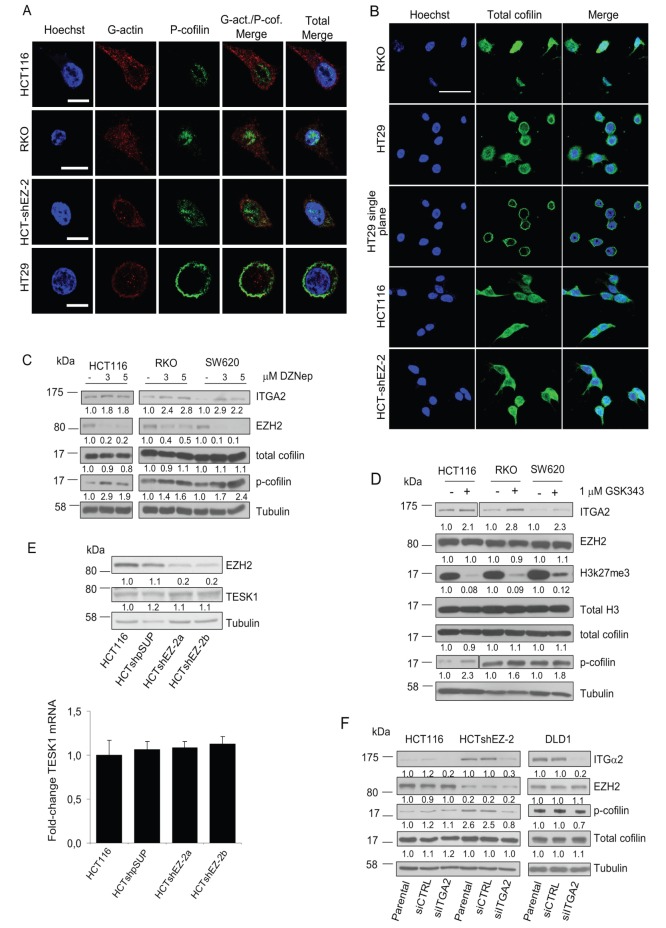
P-cofilin, cofilin and G-actin intracellular localization and regulation. A. HCT116, RKO, HCT-shEZ-2 and HT29 have been co-stained with p-cofilin (green) and G-actin (red) in order to study the intracellular localization of both proteins. Nuclei are counterstained with hoechst (blue). A merge of all three colors is also shown (merge). Scale bars 10 µm. B. Cofilin (green) staining in CRC cells. Only HT29 cells presented almost exclusive cytoplasmic localization as shown by single-plane confocal picture. Scale bars 50 µm. C. Pharmacological disruption of EZH2 protein by DZNep in CRC cell-lines with high EZH2 protein expression. D. Pharmacological inhibition of EZH2 enzymatic activity by GSK343. Low levels of H3K27me3 in treated cells indicate that GSK343 efficiently blocks EZH2 activity. E. TESK1 protein (top) and mRNA (bottom) expression analyses on CRC cells with high (HCT116 and HCT-shpSUP) and silenced (HCTsh-EZ-2a/b) EZH2 protein levels. F. siRNAs versus ITGα2 mRNA have been used to reduce ITGα2 protein expression. The effects of ITGα2 silencing on cofilin and p-cofilin were analyzed using WB. Numbers below blots indicate band quantification which was performed using a dedicated software and Tubulin expression as reference loading control. For p-cofilin quantification in HCT116 and HCT-shEZ-2 cells all values have been normalized with respect to the p-cofilin value of untransfected HCT116. All WB experiments have been repeated at least two times and representative blots are shown.

To further validate p-cofilin western blot data and confirm that p-cofilin does not contribute to cell migration when localized in the nucleus, HCT116, HCTsh-EZ-2, HT29 (highest WB p-cofilin level) and RKO (low WB p-cofilin level) cell lines were analyzed with confocal microscope by co-staining p-cofilin with G-actin and separately with total-cofilin. Fig. 2A shows that in HT29 cells p-cofilin staining is high compared to HCT116, HCT-shEZ-2 and RKO, confirming WB data. Moreover, p-cofilin in HT29 cells was exclusively detected in the cytoplasm unlike HCT116, HCTshEZ-2 and RKO cell lines. In HT29, the same cytoplasmic pattern was also detected for total-cofilin (HT29 single plane picture in Fig. 2B). Interestingly, in HCT116, HCT-shEZ-2 and RKO cells total cofilin was mainly localized in the cytoplasm unlike p-cofilin (Figs. 1E and 2B). Concerning G-actin, co-staining experiments pointed out that G-actin is localized in the cytoplasm as well as in the nucleus of all cell lines. We observed that nuclear p-cofilin rarely co-localize with G-actin in HCT116 and RKO cells, proving that p-cofilin is not able to bind G-actin in the nucleus. Furthermore, though HCT-shEZ-2 cells derived from HCT116 they show p-cofilin also in the cytoplasm similarly to HT29 cells characterized by low migratory capacity (Fig. 2A).

The relation between EZH2 and p-cofilin was also studied by blocking the methyl-transferase activity of EZH2 with two EZH2 inhibitors 3-Deazaneplanocin A (DZNep) and GSK-343 [26], [27] in cell lines where EZH2 is highly expressed: HCT116, SW620 and RKO. DZNep treatment disrupted EZH2 protein in all cell lines resulting in p-cofilin increment without affecting the total cofilin expression (Fig. 2C). Similar results were obtained with GSK-343 treatment, that efficiently blocked the enzymatic function of EZH2, since a remarkable global reduction of H3K27me3 mark was observed in treated cells, without affecting EZH2 and cofilin expression levels, despite cofilin phosphorylation (Fig. 2D).

### The EZH2-target-gene ITGα2 in part controls cofilin phosphorylation/de-phosphorylation in CRC cell lines

By chromatin immuno-precipitation (Chip) we previously demonstrated [17] that EZH2 hyper-methylates the lysine 27 of histone H3 at ITGα2 gene promoter causing epigenetic gene-repression (Fig. 1B). Interestingly, TESK1, a kinase able to phosphorylate cofilin at serine 3 and thereby able to regulate its activity, can be activated by integrin-mediated signaling [8], [28] which proposes ITGα2 pathway as a candidate for further investigation. The de-repressed signaling activity of ITGα2 through TESK1 may be responsible, at least in part, for the enhancement of p-cofilin in EZH2-silenced cells. To verify this hypothesis, first Real-Time PCR and WB analyses were performed to verify the expression of TESK1 in the CRC cell line system used for the study. It is shown (Fig. 2E) that TESK1 was expressed in HCT116 cells and that silencing of EZH2 did not affect its expression since no changes of TESK1 mRNA and protein levels have been detected among control and EZH2-silinced cells. Then, to address the role of ITGα2 on cofilin pathway, small interfering RNA oligos versus ITGα2 (siITGα2) were used to reduce ITGα2 expression in HCTshEZ-2 and HCT116 control cells. Interestingly, reduction of ITGα2 protein level negatively affected p-cofilin mainly on HCTshEZ-2 cells (Fig. 2E). Comparable results were obtained also after treatment of DLD1 cells with siITGα2. DLD1 express high levels of ITGα2 protein as compared to HCT116 (Fig. 2E) [17].

Cell treatment with the EZH2 inhibitors provided further evidence for the novel proposed epigenetic mechanism of p-cofilin regulation. As shown in Fig. 2C and 2D, DZNep and GSK-343 treatments efficiently de-repressed ITGα2 gene in HCT116, RKO and SW620 cells, that are characterized by low ITGα2 expression [17]. Indeed, EZH2 inhibitor treatments resulted in a remarkable increment of p-cofilin in HCT116 and SW620 cells, and had less, but still considerable effect on p-cofilin levels in RKO cells.

### Three-dimensional cytoskeleton organization and migration ability of CRC cell lines are controlled by cofilin phosphorylation

**Figure 3:**
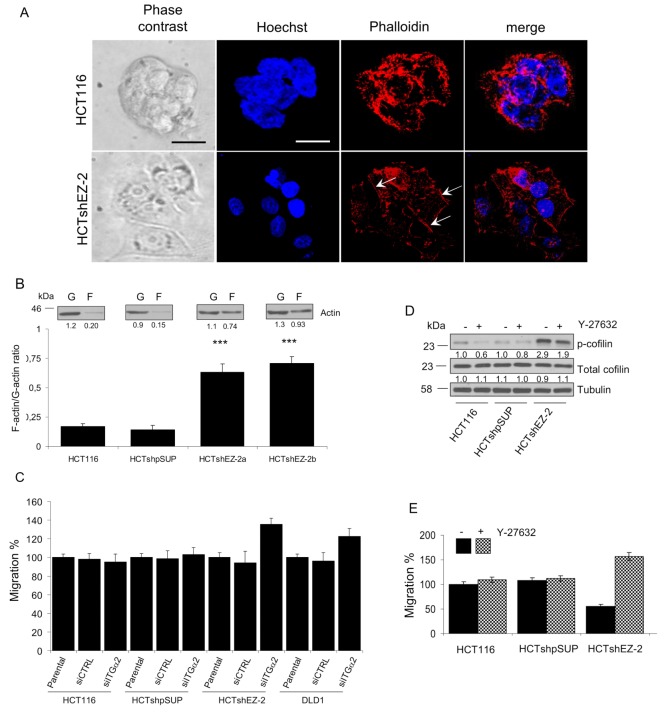
EZH2/ΙΤGα2-regulated cofilin activity controls cell morphology and migration. A. F-actin was stained with phalloin conjugated to a fluorophore (red) in HCT116 and HCTshEZ-2 cells cultured in matrigel. Representative phase contrast microscopy pictures of the same cultures are also presented for each cell line. Arrows indicate long F-actin molecules. Scale bar 20 µm. B. Analysis of relative amount of F- and G-actin in CRC cells with high (HCT116 and HCT-shpSUP) and silenced (HCTsh-EZ-2a/b) EZH2 protein levels. Intensity of WB bands has been quantified and the ratio is calculated in arbitrary units. Representative blot of F- and G-actin is also shown for each cell line. Student T-test was used to evaluate the statistic significance (*** = p-value<0,01) between HCTsh-EZ-2a/b cells and HCT116 cells. C. Migration ability of CRC cell lines transfected with siITGα2. D. WB analysis of p-cofilin levels in CRC cells with high (HCT116 and HCT-shpSUP) and silenced (HCTsh-EZ-2) EZH2 protein treated with ROCK1 inhibitor (Y27632) compared to the untreated cells. E. Migration ability of CRC cell lines treated with ROCK1 inhibitor (Y27632) compared to the untreated cells.

The morphologic changes that occur when HCTshEZ-2 cells are seeded on matrigel (three-dimensional culture, 3D) indicate that cofilin pathway may also be responsible for adhesion properties crucial for metastatic cell spread. HCT116 and HCTshEZ-2 cells cultured in 3D have been stained with phalloidin, that binds F-actin, conjugated to a fluorescent chemical compound and analyzed with fluorescent confocal microscopy (Fig. 3A). In HCTshEZ-2 cells inactive p-cofilin stabilizes actin structures necessary for the interaction with the ECM. In fact, after 3 days on matrigel, HCTshEZ-2 cells showed a flat cell-body, clearly visible nuclei and, more importantly, a single cell monolayer with well visible actin filaments (arrows in Fig. 3A). In contrast, HCT116 cells did not show high levels of F-actin and cells were able to grow as a tumour sphere.

To better verify the redistribution of actin filaments in HCTshEZ-2 cells, G-actin and F-actin were differentially isolated from HCTshEZ-2 cells as well as from control cells grown in standard 2D culture. As clearly shown in Fig. 3B F-actin levels in HCTshEZ-2 cells were much more abundant than in control HCT116 cells. Indeed, the average F-actin/G-actin ratio was approximately 3.5 times higher in HCTshEZ-2 cells as compared to the controls. Finally, to prove that aberrant inactivation of the ITGα2-cofilin axis, mediated by EZH2, increases tumour cell migration we measured migratory ability of HCT116, HCTshSUP, HCTshEZ-2 and DLD1 cells after ITGα2 silencing. As already shown in Fig. 2D and discussed above, siITGα2 efficiently reduced the expression of ITGα2 in all cell lines analyzed with consequent reduction of cofilin phosphorylation that exacerbates the F-actin/G-actin turnover. Notably, siITGα2 mainly on HCTshEZ-2 and DLD1 cells increased migration capacity by roughly 25% and 12% respectively, confirming that the EZH2-silenced ITGα2-cofilin axis is important for cancer cell locomotion (Fig. 3C). Comparable results have been obtained also with wound healing assay (S4 Fig.).

The pivotal role of p-cofilin in actin dynamics and cell migration was also confirmed by a pharmacological inhibition of ROCK1 activity, which does not involve manipulation of ITGα2 expression. Specifically, we used ROCK1 inhibitor Y-27632 to block cofilin phosphorylation [29]. ROCK1 inhibitor reduced p-cofilin in HCT116, HCTshpSUP and HCTshEZ-2 cell lines (Fig. 3D). Reduction of p-cofilin enhanced the fraction of active cofilin that was translated into a gain of migration capability mainly on HCTshEZ-2 (Fig. 3E). These results prove that in CRC cells the ratio p-cofilin/cofilin is crucial for cancer-cell spread and that ITGα2 can regulate cofilin also through pathways that involve ROCK1.

## Discussion

Different mechanisms have been described to explain the pro-metastatic activity of the histone methyltransferase EZH2 in various cancer types [30]–[32], but little is known about its role regarding CRC. In the present study, we have investigated this issue by using a loss-of-function approach to evaluate the role of the histone modifier EZH2 in CRC cell migration. Specifically, we took advantage of the possibility to further characterize a CRC cell line (HCT-shEZ-2) where EZH2 is permanently silenced and of a new compound able to specifically inhibit EZH2 methyltransferase activity, named GSK-343, as well as a compound able to disrupt the PRC2 complex, named DZNep. It is shown that in this cell line EZH2 epigenetically controls cell locomotion by keeping cofilin steadily active. Indeed, after genetic and pharmacological inhibition of EZH2, cofilin phosphorylation at serine 3 was increased. The serine-3-phosphorylated cofilin is inactive. As a consequence of cofilin inactivation, invadopodia assembling at the leading edge of migrating cells as well as disassembling in the back-cell-side cannot be modulated, resulting in a reduction of cell locomotion [3], [6]. This may explain the remarkable reduction cell mobility of HCT-shEZ-2 as compared to the parental HCT116 cells [17]. The direct silencing action of EZH2 on up-stream components of cofilin pathway was excluded. Indeed, no changes in protein expression and activity have been detected by WB, GST-PAK pull down and siRNA assays for, CDC42, RAC1, RhoA, ROCK1 as well as total cofilin in HCT-shEZ-2 cells with respect to control cells. To confirm the hypothesis that EZH2 regulates cell migration via an alternative biological axis and not by repressing Rho GTPase family components, we sought to investigate on the role of ITGα2 gene. In fact, ITGα2 is directly regulated by EZH2 [17] and potentially can act on cofilin pathway. Accordingly, previous studies have shown that TESK1 is able to phosphorylate cofilin at serine 3 and notably TESK1 can be activated by integrin-mediated signaling [8], [28]. Quantitative PCR and WB analysis revealed that TESK1 is expressed in HCT116 cell line and that EZH2 silencing did not change TESK1 expression level. It is further demonstrated that ITGα2 is the major regulator of cofilin phosphorylation by silencing ITGα2 protein in HCT116, HCT-shEZ-2 and in DLD1 cells. Indeed, after ITGα2 silencing p-cofilin levels are reduced, proving a direct relation among ITGα2 and p-cofilin possibly mediated by TESK1, though we cannot exclude the involvement of other kinases targeted by integrins. Nevertheless, based on the above evidence, we propose that EZH2 through the epigenetic silencing of ITGα2 gene is able to control cytoskeleton remodeling and cell-migration. F-actin disassembly, for example, is necessary for the protrusion of lamellipodia during fibroblast migration [33] and the activity of cofilin, which generates new barbed ends, in association with the Arp2/3 complex is crucial to stimulate branching of actin filaments in invasive tumours cells [34]. A constitutive cofilin activation, as found in HCT116, may increase the rate of F-actin severing, thus allowing cancer cells to rapidly activate a motility cycle which is translated in enhanced migratory capacity. In HCT-shEZ-2 this motility cycle is strongly reduced because of over-phosphorylation of cofilin which results in F-actin stabilization, as well as reduction of cell mobility. Furthermore, we observe a partial nuclear translocation of cofilin when it is phosphorylated/inactive in cell lines (HCT116 and RKO), characterized by an invasive phenotype. HCT116 and RKO cells present low protein expression of the epithelial marker E-cadherin and high protein expression of the mesenchymal marker N-cadherin, a pattern of markers which characterizes highly migrating phenotypes. By contrast, in the poorly migrating HT29 cell line (with high E-cadherin and low N-cadherin) [17], both cofilin and p-cofilin present a clear cytoplasmic staining. Even though cofilin presents a nuclear localization signal (NLS), the precise role of both cofilin and p-cofilin in the nucleus is still under investigation. It has been proposed that as actin has no NLS but is involved in gene expression, the binding with cofilin can allow an efficient actin nuclear transport [3]. In addition, a novel role for nuclear cofilin has recently been reported in breast cancer by Madak-Erdogan et al. [35]. Cofilin is able to interact with the protein kinase ERK5 and both are recruited into the nucleus where they bind the transcription start site (TSS) of estrogen-stimulated genes upon hormone treatment of ERα-containing breast cancer cells. This nuclear translocation, though, is absent in breast cancer cells that lack ERα, so ERK5 and cofilin remain outside the nucleus increasing cell motility and invasiveness [35]. However, in Madak-Erdogan et al. study only the intracellular localization of cofilin was analyzed and no information is provided about the phosphorylation state of cofilin during cytoplasmic-nuclear translocation. In our study, co-staining experiments showed that p-cofilin rarely co-localizes with G-actin in the nucleus, confirming the idea that p-cofilin in the nucleus does not promote cell migration but it may be involved in other functional activities.

Intriguing is also the fact that Caco-2 cells have extremely low levels of p-cofilin as well as low EZH2 expression with respect to the other cell lines such as HCT116, RKO and SW620. Caco-2 cells present a mild tumour phenotype, no ability to move, low proliferation rate and express several factors typically found in mature/well-differentiated enterocytes [17] that apparently contrast with active cofilin. A possible explanation is that cofilin in Caco-2 cells may be inactivated by other mechanisms. In fact, phosphorylation at serine 3 of cofilin protein is not the only way to inactivate its biological activity. Cofilin is also inhibited by binding phophatidylinositol 4,5-bisphosphate [36], cortactin [37] and by the increasing of intracellular ph [38]. We cannot exclude that one of these mechanisms is keeping the cofilin pathway idle in Caco-2 cell line.

We also investigated the morphological and migratory properties of CRC cells that are controlled by the axis EZH2/ITGα2/p-cofilin. By using matrigel (3D culture) remarkable morphological changes have been observed in HCTshEZ-2 as compared to the control HCT116 cells, that can be ascribed to the stabilization and to the gain of F-actin in HCTshEZ-2. In addition, by reducing p-cofilin via ITGα2 silencing and by treatment with ROCK1 inhibitor it has been further proved that the pro-migratory activity of EZH2 in CRC takes part in regulatory mechanisms that control cell-shape and cell-protrusions and that phosphorylation of cofilin is crucial for cancer cell-migration.

**Figure 4:**
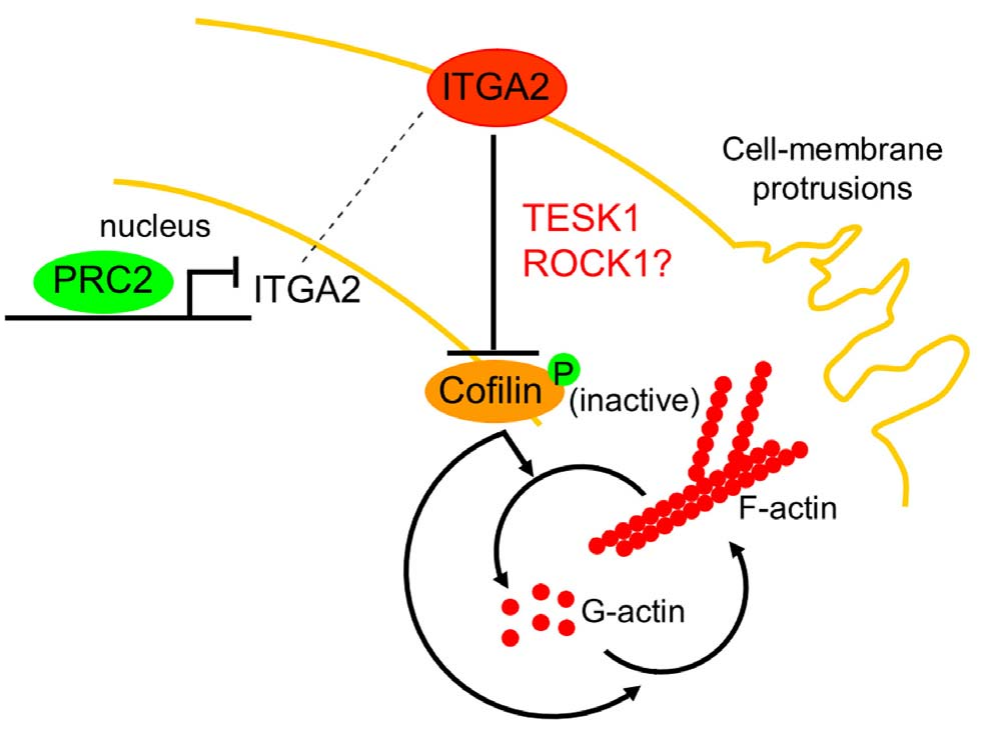
Proposed model of cofilin regulation by EZH2-ITGα2 axis. EZH2 via hyper methylation of lysine 27 of histone H3 at ITGα2 promoter impairs ITGα2 gene-expression. The absence of ITGα2 signaling results in cofilin over-activation and consequently actin dynamics is increased.

Taking together all results, we propose a model to explain how a sub-set of CRC cells may acquire migratory properties through an alternative non-conventional and novel epigenetically regulated pathway (Fig. 4). The model proposed in this study, even though derives from evidence gathered from a limited number of CRC cell lines, integrates epigenetic gene-silencing via histone post-translation modifications, de-regulation of actin filament dynamics, as well as anchorage-mediated signaling. It is known that movement of normal cells is also controlled by contact inhibition, which largely depends on adhesion molecules such as integrins, that prevents aberrant cell migration [39]. In pathological conditions, over-expression of EZH2 results in ITGα2 silencing. Consequently, cell-matrix interactions are reduced and integrin-signaling activity impaired, followed by reduced TESK1 and/or ROCK1 kinase activity. Although further studies are needed to clarify the involvement and the precise role of these kinases, it can be proposed that these deficiencies enhance mobility of CRC cells. Indeed, EZH2 enhances the severing activity of cofilin and, because of ITGα2 repression, cells are weakly anchored to the ECM. These findings also propose novel epigenetic mechanisms and factors that may provide new anti-metastatic options for the treatment of metastatic CRC malignances.

## Supporting Information

S1 Fig **GST-PAK Pull-down assay.** Activation status of RAC1 and CDC42 was evaluated by measuring the levels of RAC1-GTP and CDC42-GTP in each cell line. Numbers below RAC-GTP and CDC42-GTP blots have been calculated over the amount of crude protein extracts (Total) used in the pull-down assay.
(EPS)

S2 Fig **A. RhoA silencing assay.** RhoA protein was transiently silenced in HCT116, HCTshpSUP, HCTshEZ-2a and HCTshEZ-2b cell lines using siRNA technology and p-cofilin levels were evaluated by WB. **B.** Transient depletion of EZH2 with two different siRNA oligos increases cofilin phosphorylation similarly to stable silencing also in RKO CRC cell line. All WB experiments have been repeated at least three times, representative blots and quantifications are shown.
(EPS)

S3 Fig **Correlation analysis of EZH2 protein levels versus cofilin phosphorylation levels.** Each point represents a single cell line and is identified by an ordered pair of values that represent the absolute intensity of p-cofilin bands (x-axis) and the absolute intensity of EZH2 bands (y-axis) both from Fig. 1F. The equation of the straight line is also showed with a correlation coefficient (R) of −0.7532.
(EPS)

S4 Fig **Wound healing assay on HCT116, HCTshpSUP, HCTshEZ-2a and HCTshEZ-2b cell lines treated with siITGα2.** The above mentioned cell lines have been transfected with siITGα2. Twenty-four hours after transfection a scratch was performed on the culture plates and representative pictures were taken. Scratches have been photographed again after 24 hours to evaluate the migration.
(EPS)
